# Antibiotic resistance, biofilm formation, and biofilm-associated genes among *Stenotrophomonas maltophilia* clinical isolates

**DOI:** 10.1186/s13104-021-05567-y

**Published:** 2021-04-20

**Authors:** Narjess Bostanghadiri, Abdollah Ardebili, Zohreh Ghalavand, Samane Teymouri, Mahsa Mirzarazi, Mehdi Goudarzi, Ehsan Ghasemi, Ali Hashemi

**Affiliations:** 1 Department of Microbiology, School of Medicine, Iran University of Medical Sciences, Tehran, Iran; 2 Laboratory Sciences Research Center, Golestan University of Medical Sciences, Gorgan, Iran; 3 Department of Microbiology, Faculty of Medicine, Golestan University of Medical Sciences, Gorgan, Iran; 4 Department of Microbiology, School of Medicine, Shahid Beheshti University of Medical Sciences, Tehran, Iran; 5 Student Research Committee, Department of Medical Biotechnology, School of Advanced Technologies in Medicine, Shahid Beheshti University of Medical Sciences, Tehran, Iran; 6 Abadan School of Medical Sciences, Abadan, Iran

**Keywords:** *Stenotrophomonas maltophilia*, Antibiotic resistance, Biofilm, Biofilm formation genes

## Abstract

**Objective:**

The purpose of the present study was to investigate the antimicrobial susceptibility pattern, biofilm production, and the presence of biofilm genes among the *S. maltophilia* clinical isolates. A total of 85 clinical isolates of *S. maltophilia* were collected from patients referred to several hospitals. Susceptibility to antibiotics was investigated by disc diffusion method according to the guidelines of the Clinical and Laboratory Standards Institute (CLSI). By the crystal violet staining method, the capability of biofilm formation was examined. The genes associated with biofilm production were investigated by the PCR-sequencing techniques.

**Results:**

All isolates were resistant to doripenem, imipenem, and meropenem. Minocycline, trimethoprim/sulfamethoxazole and levofloxacin exhibited the highest susceptibility of 100%, 97.65%, and 95.29%, respectively. The results of crystal violet staining assay showed that all isolates (100%) form biofilm. Moreover, 24 (28.23%), 32 (37.65%), and 29 (34.12%) of isolates were categorized as weak, moderate, and strong biofilm producers, respectively. Biofilm genes including *rpfF*, *spgM* and *rmlA* had an overall prevalence of 89.41% (76/85), 100% (85/85) and 84.71% (72/85), respectively. Rational prescribing of antibiotics and implementation of infection control protocols are necessary to prevent further infection and development of antimicrobial resistance. Combination strategies based on the appropriate antibiotics along with anti-biofilm agents can also be selected to eliminate biofilm-associated infections.

## Introduction

*Stenotrophomonas maltophilia*, previously known as *Pseudomonas maltophilia* or *Xanthomonas maltophilia*, has become nowadays a major opportunistic pathogen in hospitalized or immunocompromised patients worldwide [1]. *This organism is the most prevalent non-fermenting Gram-negative bacilli in clinical laboratories* after *P. aeruginosa* and *Acinetobacter baumannii* [2]. In addition, it is known to causes severe infections such as acute exacerbations of chronic obstructive pulmonary disease (COPD), pneumonia, bacteremia, sepsis, bone, and joint infections, eye infections, endocarditis, and meningitis [3, 4]. *S. maltophilia* isolates show resistance to a variety of antibacterial agents, with various types of antimicrobial resistance mechanisms [5–7], leading to a great challenge for physicians and clinical microbiologists to manage related infections [8, 9].

A prominent feature of *S. maltophilia* is its capability to adhere to abiotic surfaces, host tissues and biofilm formation [10, 11]. *S. maltophilia* has been identified on the surfaces of biomaterials used in prosthetic devices, intravenous cannula, dental unit waterlines and nebulizers [12–14]. The biofilm-forming capacity of *S. maltophilia* has increasingly been accepted as an important virulence factor and is thought to play a significant role in the persistence of *S. maltophilia* infections in hospital settings [10, 15–19] The molecular mechanisms of biofilm formation in *S. maltophilia* is poorly investigated [18, 19]. Several genes are associated with biofilm-formation. The *spgM* gene encodes a bifunctional enzyme with both phosphoglucomutase and phosphomannomutase activities that is involved in LPS biosynthesis, playing an important role in biofilm formation [18, 20]. Mutation in *spgM gene*, may cause fewer LPS production and shorter O polysaccharide chains [21]. The *rmlA* gene encodes glucose-1-phosphate thymidyltransferas that is involved in LPS/EPS-coupled biosynthetic pathway It is reported that mutations in *rmlA* and *rpfF* genes resulted in reduced biofilm formation in *S. maltophilia* [4, 19]. The *rpfF* gene, encoding the DSF (diffusible signal factor) synthase regulates the virulence expression, such as motility, extracellular proteases, LPS, and biofilm production. RpfF protein has some amino acid sequences similar to enoyl coenzyme A hydratases [19].

Considering the potential of biofilm in increasing antimicrobial resistance and subsequently, the increased rates of chronic infections caused by *S. maltophilia*, identification of the isolates with such factor will be benefit to better understand the pathogenesis of the organism. The aim of this survey was to investigate the pattern of antibiotic susceptibility, the ability of biofilm production, and the presence of biofilm-related genes in clinical *S. maltophilia* isolates.

## Main text

### Methods

#### Bacterial isolates and species identification

*S. maltophilia* isolates included in this study were originated from different clinical samples of patients admitted at selected hospitals in Tehran, Iran from January 2018 to January 2019. All of the isolates were identified by standard microbiological and biochemical methods, including catalase and oxidase tests, reactions in media, such as triple sugar iron agar, deoxyribonuclease test agar, and SIM (Merck company, Germany). *S. maltophilia* isolates were then confirmed by the PCR amplification of the 16S rRNA gene and sequencing (Table 1). All isolates were stored in Luria–Bertani (LB) liquid medium (Merck company-Germany) containing 15% glycerol at − 80 °C. *Escherichia coli* ATCC 25,922 and *S. maltophilia* ATCC 13,637 were used as the quality control strains.

**Table 1:** Oligonucleotide primers used in this study

Genes	Sequences (5’→3’)	Target	References
*16srRNA-F*	AGTTCGCATCGTTTAGGG	16 s RNA	[2]
*16srRNA-R*	ACGGCAGCACAGAAGAGC		
*spgM-F*	ATACCGGGGTGCGTTGAC	spgM	[18]
*spgM-R*	CATCTGCATGTGGATCTCGT		
*rpfF-F*	CACGACAGTACAGGGGACC	rpfF	[18]
*rpfF-R*	GGCAGGAATGCGTTGG		
*rmlA-F*	CGGAAAAGCAGAACATCG	rmlA	[3]
*rmlA-R*	GCAACTTGGTTTCAATCACTT		

#### Antimicrobial susceptibility testing

The antibiotic sensitivity pattern of *S. maltophilia* isolates was evaluated by the Kirby-Bauer disc diffusion method as described by the Clinical and Laboratory Standards Institute (CLSI) [22]. Antibiotic discs used for susceptibility testing were levofloxacin (5 μg), minocycline (30 μg), imipenem (10 μg), meropenem (10 μg), doripenem (10 μg), sulfamethoxazole/trimethoprim (SMX/TMP) (1.25/23.75 μg), tetracycline (30 μg), and ceftazidime (30 μg) (MAST Diagnostics, Merseyside, UK). Control strains of *E. coli* ATCC 25922 and *E. coli* ATCC 35218 were used for susceptibility testing.

#### DNA preparation

*S. maltophilia* isolates were cultivated on Columbia agar medium (bioMérieux Italia S.p.A-Italy) supplemented with 5% sheep blood and incubated at 37 °C for 24 h. The DNA samples were extracted from the grown colonies of each isolate with high pure PCR Template Preparation Kit (Roche company-Germany). The total DNA concentration was evaluated by Nanodrop (WPA Biowave II Nanospectrophotometer-USA).

#### PCR-sequencing techniques

The presence of biofilm-encoding genes, including *rpfF*, *spgM*, and *rmlA* was investigated in *S. maltophilia* isolates by PCR technique using the specific primers (Table 1). PCRs were performed on a thermal cycler (Eppendorf, Master Cycler Gradient- Germany) in 25-μl reaction volumes with 1 μl (20 ng) of DNA template, 1 × PCR buffer, 12.5 μl of 2 × Master Mix (SinaClon-Iran), 3 mmol/L MgCl_2_, 0.4 mmol/L dNTPs, 9.5 μl of sterile distilled water, 1 μl of 10 pmol of each primer, and 0.08 IU of *Taq* DNA polymerase. PCR conditions were under the following: denaturation at 95 °C for 10 min, and then 36 cycles at 94 °C for 60 s, annealing at 52–60 °C, depending to the primers for each gene, for 60 s, and extension at 72 °C for 60 s, followed by a final extension at 72 °C for 5 min. PCR products were electrophoresed by 1.2–1.5% agarose gel, visualized by DNA Safe staining, and then photographed under UV light.

The amplicons were purified using a PCR purification Kit (BioFact Co., South Korea), and then sequenced by an ABI PRISM 3700 sequencer (Applied Biosystems Inc., USA). The nucleotide sequences were analyzed using FinchTV software and comparisons were made using the NCBI BLAST program (http://www.ncbi.nlm.nih.gov/BLAST/).

#### Biofilm formation assay

Biofilm formation by *S. maltophilia* was evaluated using crystal violet staining method as previously described by Stepanović et al. [23]. All experiments were run in triplicate. An overnight culture of isolates was adjusted to the turbidity of a 1.0 McFarland standard. Suspensions were diluted at a ratio of 1:100 in 200 ml tryptic soy broth (TSB) (Merck, Darmstadt- Germany) containing 1% glucose and, were dispensed to the sterile flat-bottomed 96- well polystyrene microplates (JET Biofil, Guangzhou, China). After 24 h of incubation at 37 °C, microplates were washed three times with sterile phosphate-buffered saline (PBS, pH 7.3). Adherent biofilms were fixed for 1 h at 60 °C, stained by 200 µl Hucker modified crystal violet (Sigma Chemical Company- USA) for 5 min at room temperature and then rinsed with water and allowed to dry. Biofilm samples were destained with 200 ml 33% glacial acetic acid for 15 min. The optical density (OD) was measured at 492 nm using a microtiter plate reader (BioTek, Bad Friedrichshall, Germany). A cut-off value (ODc) was established. It is defined as three standard deviations (SD) above the mean OD of the negative control: ODc = average OD of negative control + (3 × SD of negative control). The isolates were categorized into four groups according to the following criteria: non-biofilm producer (OD < ODc), weak-biofilm producer (ODc < OD < _2 × ODc), moderate-biofilm producer (2 × ODc < OD < 4 × ODc), and strong-biofilm producer (4 × ODc < OD).

### Statistical analysis

The statistical analysis of data was performed with statistical software SPSS, 20.0 (SPSS Inc., Chicago, IL, USA). Chi-squared test was used to determine the association between categorical variables. A *p*- value ≤ 0.05 was considered statistically significant.

### Results

#### Patients and bacterial isolates

During one-year period, 85 *S. maltophilia* isolates were gathered from several health centers in Tehran, Iran. Among them, 49 isolates were from males and 36 isolates from females (male: female ratio = 1.36). Most of the *S. maltophilia* (90.03%) were isolated from the blood, while the rest (9.97%) were from the cough swabs. The range of patients' age was from 2 months to 85 years.

#### Antimicrobial susceptibility

The results of susceptibility testing on planktonic cells showed that approximately 100%, 96%, 96%, 36.58%, 2.35% of the *S. maltophilia* isolates were resistant to imipenem, doripenem, meropenem, ceftazidime and SMX/TMP-, respectively. Levofloxacin and minocycline (95.29% and 100% susceptibile, respectively) exhibited the highest activity against *S. maltophilia*, with a rate of (Table 2).

**Table 2:** Antibiotic susceptibility of *S. maltophilia* clinical isolates (*n* = 85)

Antimicrobial agents	Antimicrobial susceptibility pattern, n (%)		
	Susceptible	Intermediate	Resistant
Imipenem	–	–	85 (100%)
Meropenem	–	–	85 (100%)
Doripenem	–	–	85 (100%)
Ceftazidime	24 (28.24%)	–	61 (75.72%)
Minocycline	85 (100%)	–	–
Levofloxacin	81 (95.29%)	–	4 (4.71%)
Sulfamethoxazole/trimethoprim	83 (97.65%)	–	2 (2.35%)

#### Biofilm formation

In this study, the biofilm forming ability was assessed on polystyrene using the microtiter plate method. Biofilm phenotypes accounted for 100%, being distributed as follow: 24 isolates (28.23%) produced weak biofilm, 32 isolates (37.65%) produced moderate biofilm, and 29 isolates (34.12%) produced strong biofilm.

#### Biofilm-encoding genes

**Fig. 1:**
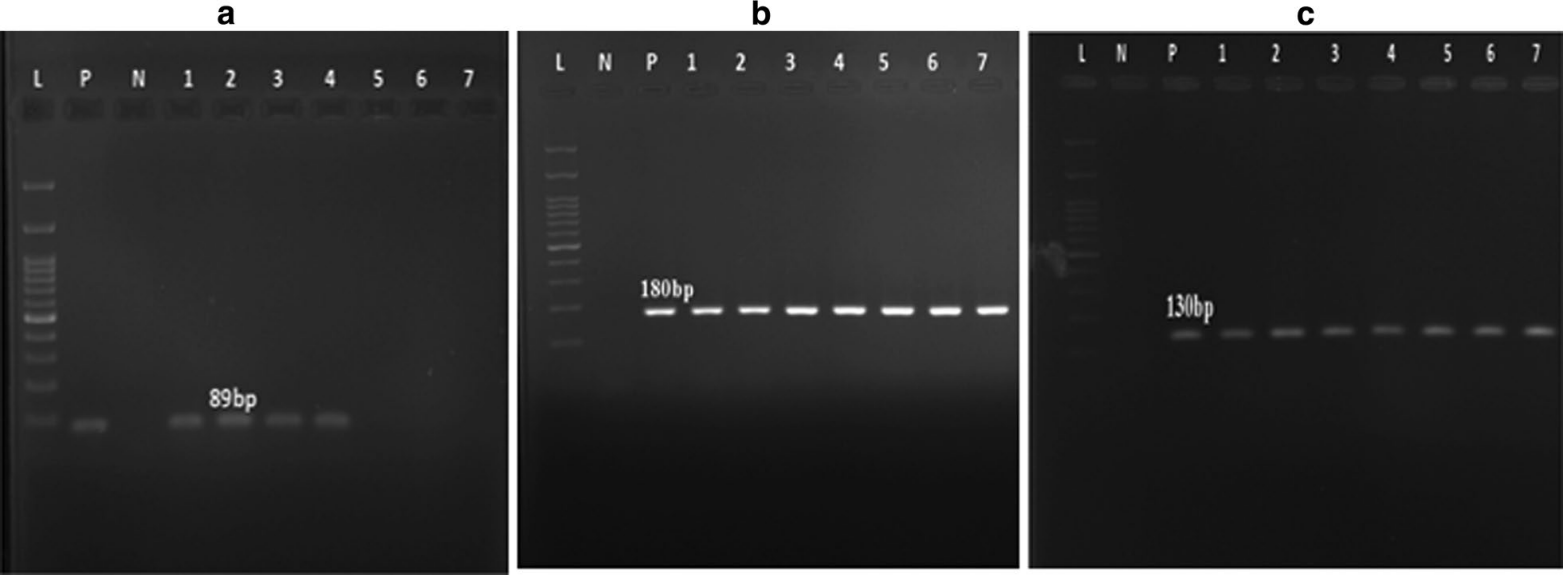
The amplification of biofilm-encoding genes in *S. maltophilia* isolates. L: 100 bp DNA ladder, P: positive control, N: negative control, Lanes 1 to 7: PCR products of the corresponding genes:** a**; *rmlA* gene,** b**; *rpfF* gene, ** c**; *spgM* gene

The frequency of biofilm-related genes among the *S. maltophilia* isolates was generally as high as 89.41%, 100%, and 84.71% for *rmlA,* *spgM* and *rpfF* genes, respectively (Fig. 1). Among them, 63 isolates carried all three genes studied. *S. maltophilia* isolates with *spgM* + /*rpfF* + /*rmlA* + genotype showed strong or moderate biofilm-producer phenotype.

### Discussion

*S. maltophilia* is increasingly identified as a cause of nosocomial infections, especially among immunosuppressed patients [24]. Treatment of infections caused by this pathogen is a problem for clinicians because of its resistance to a broad array of antimicrobial drugs [25]. In the present study, all isolates were resistant to carbapenems (*p* ≤ 0.001). Similarly, a previous study showed that resistance rates to imipenem and meropenem in *S. maltophilia* were 100% and 92.4%, respectively [26]. Moreover, our results showed a susceptibility rate of 28.24% to ceftazidime. A study by Farrell et al. showed that susceptibility of *S. maltophilia* against ceftazidime was 32.51% in Latin America, North America, Asian-Pacific, and Europe [26]. In contrast, Jamali et al. reported the susceptibility rate of 82% against this drug [27]. In our study, 100% and 95.29% of *S. maltophilia* were susceptible to minocycline and levofloxacin, respectively. Duan et al. showed also the susceptibility rates of 100% and 95.7% to minocycline and levofloxacin, respectively [28]. These findings indicate that such antibiotics serve as effective agents for treatment of *S. maltophilia* infections. On the other hand, the most effective antimicrobial agent used to treat *S. maltophilia* infections is SMX/TMP [29]. In a study by Jamali et al., 5% of isolates were resistant to SMX/TMP [30]. The susceptibility rates were reported as high as 95% in several studies conducted in most regions, including Latin America, North America, Europe [30–32]. In our study, 97.65% of isolates were found to be susceptible to SMX/TMP, indicating this antibiotic has increasingly become the last resort drug for the treatment of multi-resistant *S. maltophilia* infections. However, the highest rates of resistance have been reported in isolates obtained from patients in Asian countries, such as Malaysia, Korea, and Taiwan [33]. The present study generally reveals a low frequency of antibiotic resistance among the *S. maltophilia*. However, monitoring of the antibiotic resistance trends is nesserary either geographically or over time.

All *S. maltophilia* isolates tested in this study were able to produce biofilms. In a study by Flores-Trevino et al., isolates were categorized as weak- (47.9%), moderate- (38.7%), or strong- (13.4%) biofilm producers [34]. In contrast, Gallo et al. showed that isolates were biofilm producers as weak (3%), moderate (45%), or strong (48%) [35]. From 2016 to 2017 in Iran, 47.9, 38.7, and 13.4% of out of 164 *S. maltophilia* clinical isolates were weak-, moderate-, and strong-biofilm producers, respectively [36].

Biofilms have been recognized to be involved in many chronic and intractable infections [13, 37]. The results of this study showed biofilm formation significantly correlated with ceftazidime and SMX/TMP resistance*.* Similarly, biofilm formation has been shown associated with resistance to different antibiotics, including ceftazidime, piperacillin/tazobactam, cefepime, ticacillin/clavulanic acid, aztreonam, and gentamicin [38]. more understanding of biofilm dynamics can lead to development of a effective treatment, and control, strategies for eradication of infections, improving patient care [39].

In the present study, we investigated the relationship between biofilm formation and the presence of its related genes *rpfF*, *spgM*, and *rmlA*. Sixty-three isolates of *S. maltophilia* strains had all genes studied, while only 81 strains carried the *spgM*. Overall, our results revealed that the presence of the *spgM* significantly promoted biofilm formation, in accordance to those obtained by Pompilio et al. [40]. In a study by Zhongliang Duan et al. the rates of *spgM*, *rmlA,* and *rpfF* biofilm genes were 100%, 83.7%, and 45.2%, respectively [17]. Zhuo et al. indicated that biofilm formation was considerably associated with the presence of *rpfF* and *spgM* genes [18]. Moreover, the presence of either *rpfF* or *spgM* was significantly correlated to biofilm production, although the strongest biofilm was formed when both genes were present [15]. The presence of *spgM*, *rpfF*, and *rmlA* genes improved significantly the biofilm production in our isolates.

In conclusion, although the rate of resistance to multiple antibiotics among our *S. maltophilia* isolates was relatively low, cautious antimicrobial use and high standards of infection prevention and control are needed to prevent further development of resistant isolates. Additionally, combination strategies based on the proper antibiotics with anti-biofilm agents can be used to improve the treatment of biofilm-associated infections.

## Limitations

A limitation of this study may be the lack of evaluation of expression levels of biofilm-associated genes by quantitative real-time PCR, an approach that may help to assess the role of each corresponding gene in biofilm production.

## Data Availability

The datasets generated and analyzed during this reasearch were included in the main document of this manuscript.

## Abbreviations

CLSI: Clinical and laboratory standards institute
COPD: Chronic obstructive pulmonary disease
EPS: Extracellular matrix polysaccharides
PGM: Phosphoglucomutase
TSB: Tryptic soy broth
OD: Optical density
PCR: Polymerase chain reaction
